# Effect of lactation on postpartum pelvic floor muscle regeneration in preclinical model

**DOI:** 10.1038/s44294-025-00079-7

**Published:** 2025-06-12

**Authors:** F. Boscolo Sesillo, H. Manoochehri, P. Duran, E. Zelus, K. L. Christman, M. Alperin

**Affiliations:** 1 Department of Obstetrics, Gynecology, and Reproductive Sciences, Division of Urogynecology and Reconstructive Pelvic Surgery, University of California, San Diego, San Diego, CA USA; 2 Sanford Consortium for Regenerative Medicine, La Jolla, CA USA; 3 Division of Biological Sciences, University of California, San Diego, La Jolla, CA USA; 4 Shu Chien-Gene Lay Department of Bioengineering, University of California, San Diego, La Jolla, CA USA; 5 Sanford Stem Cell Institute, La Jolla, CA USA

**Keywords:** Reproductive biology, Physiology

## Abstract

Pelvic floor muscle (PFM) recovery following childbirth is essential for preserving pelvic floor function. Despite this, the impact of parturition and lactation on pelvic muscle stem cells (MuSCs), indispensable for skeletal muscle maintenance and regeneration, remains unknown. We determined that vaginal delivery does not cause mechanical injury of the rat PFMs, enabling us to uncouple the effects of lactation on muscle homeostasis from PFM regeneration following simulated birth injury (SBI). Tibialis anterior (TA) served as non-pelvic control. This novel study demonstrates that in the absence of birth injury, lactation blocks MuSC proliferation in PFM and TA, suggesting that postpartum systemic milieu affects MuSCs in pelvic and non-pelvic muscles. In contrast, SBI negated the inhibitory effect of lactation on MuSCs in PFM but not in TA, indicating that local signals released by the injured muscle overcome systemic inhibitory effects of lactation, which persist in muscles remote from the site of injury.

## Introduction

Lactation is a unique physiological state defined by milk production that begins at the end of pregnancy and persists during the postpartum period in response to regular removal of milk and nipple stimulation during nursing^1^. Lactation has been associated with multiple neonatal and maternal health benefits, including greater maternal weight loss and reduction in body fat compared to non-lactating mothers. Triglycerides, total and LDL-cholesterol levels are also reduced with lactation^2–4^. In addition, lactating women who had gestational diabetes, have lower levels of fasting glucose and better glucose tolerance postpartum compared to the non-lactating counterparts^2^. Mothers who nurse their infants are also at a lower risk of developing cardiovascular diseases^3,5^ and metabolic syndrome compared to non-lactating mothers^4,6,7^. Despite these and other maternal benefits, breastfeeding also comes at a cost. The metabolic needs imposed by lactation are significant, as the nutrients—lipids, sugars, and proteins - are mobilized from maternal storage and redirected for milk production and secretion, impacting multiple organ systems, including skeletal muscle^2,3,8^. The high calcium content of the human milk contributes to decreased bone mineral density in lactating women with poor nutritional status. Indeed, breastfeeding has been also associated with increased risk of osteoarthritis^9^ and rheumatoid arthritis^10^. Altogether, human studies focused on the global effect of lactation are inconclusive, with disparate results likely driven by the unaccounted consequences of variable caloric intake and activity levels in the postpartum period^2,3^.

Computational models of human parturition demonstrate that during vaginal childbirth, pelvic floor muscles (PFMs), which span the pelvic outlet, are subjected to dramatic strains, stretching as much as 3 times the initial resting muscle length^11,12^. Mechanical PFM injury consequent to these supraphysiological strains and the resultant muscle dysfunction are the leading risk factors for the development of symptomatic pelvic floor disorders^13^. Pelvic floor disorders, including urinary and fecal incontinence, and pelvic organ prolapse, are morbid and costly conditions that negatively impact millions of women worldwide^13,14^. The findings of the published epidemiological studies examining the role of lactation in the development of pelvic floor disorders are conflicting. While some investigations report increased rate of urinary incontinence in women who lactated for more than 4 months, others do not find significant association between breastfeeding and pelvic floor disorders in the decade following childbirth in women with at least one vaginal delivery^15–18^. These clinical studies add invaluable data, but have multiple limitations, including participant recall bias concerning duration of breastfeeding, as well as the lack of information regarding hormonal contraception use during lactation and the resultant hormonal milieu. Importantly, the differential demographic and clinical characteristics of women who do not breastfeed relative to women who do breastfeed could predispose non-lactating group to pelvic floor disorders, negating potential protective effect of not breastfeeding on the postpartum recovery of pelvic soft tissues^17^.

While significant strides have been made to better understand the impact of pregnancy, delivery, and breastfeeding on women’s health over the lifespan, the effect of lactation on PFMs, integral to the proper function of the female pelvic floor, has not been elucidated to date. The major players in skeletal muscle plasticity and regeneration are resident muscle stem cells (MuSCs), localized between the basal lamina and the sarcolemma^19,20^. These cells are quiescent in homeostatic conditions, but become activated, proliferate, and differentiate under the influence of variable physiological cues and after muscle injury enabling tissue repair^21^. We have previously explored PFM regeneration following simulated birth injury in a non-pregnant rat model, validated for the studies of the human PFMs^22–25^. In the current study, using a more translationally relevant pregnant rat model, we aimed to determine the impact of parturition and lactation on MuSCs and PFM regeneration in the immediate postpartum period. We hypothesized that lactation may negatively affect MuSC function and muscle regeneration in response to either hormonal changes in the early post-partum period and/or to the relocation of energy resources from the skeletal muscle towards milk production.

## Results

### Spontaneous vaginal delivery does not lead to significant injury of the rat pelvic floor muscles

**Fig. 1:**
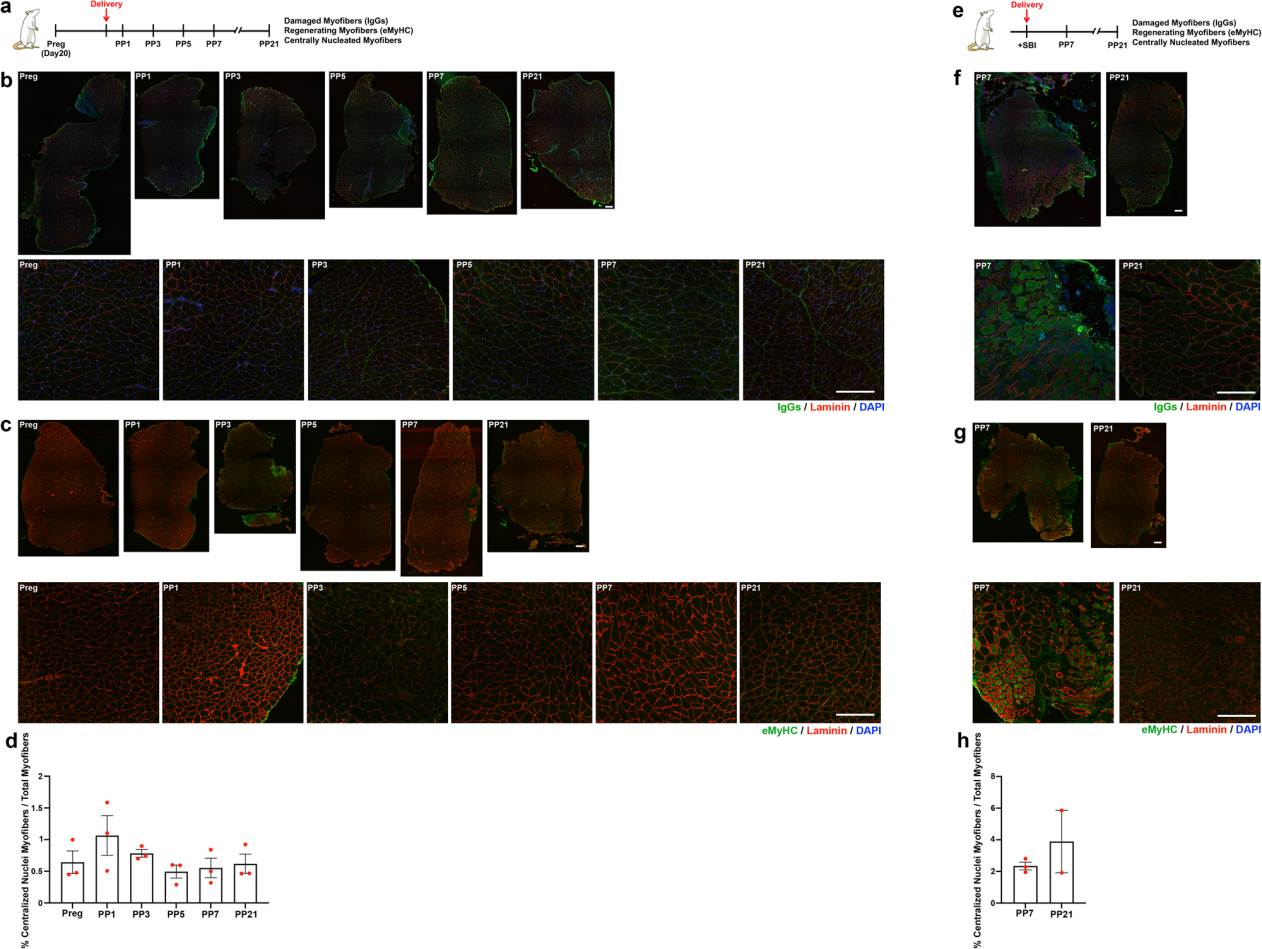
Spontaneous vaginal delivery does not cause myofiber injury of the rat pelvic floor muscles in contrast to simulated birth injury. **a** Experimental summary for (**b**, **d**). **b** Immunofluorescent staining of pubocaudalis (PCa) from pregnant (*n* = 3), postpartum (PP)1 (*n* = 3), PP3 (*n* = 3), PP5 (*n* = 3), PP7 (*n* = 3), PP21 (*n* = 3) animals for IgG. Top, full muscle section image; bottom, zoomed in images. IgG in green, laminin in red, and DAPI in blue. Scale Bar: 100 mm. **c** Immunofluorescent staining of PCa from pregnant (*n* = 3), PP1 (*n* = 3), PP3 (*n* = 3), PP5 (*n* = 3), PP7 (*n* = 3), PP21 (*n* = 3) animals for eMyHC. Top, full muscle section image; bottom, zoomed in images. eMyHC in green, laminin in red, and DAPI in blue. Scale Bar: 100 mm. **d** Bar graph representing the percentage of centralized nuclei in tissue samples from pregnant, PP1, PP3, PP5, PP7, PP21. **e** Experimental summary for (**f**, **h**). **f** Immunofluorescent staining of PCa procured from animals exposed to SVD + SBI on PP7 (*n* = 3) and PP21 (*n* = 3) against IgGs. Top, full muscle section image; bottom, zoomed in images. IgG in green, laminin in red, and DAPI in blue. Scale Bar: 100 mm. **g** Immunofluorescent staining of PCa procured from animals exposed to SVD + SBI on PP7 (*n* = 3) and PP21 (*n* = 3) against eMyHC. Top, full muscle section image; bottom, zoomed in images. eMyHC in green, laminin in red, and DAPI in blue. Scale Bar: 100 mm. **h** Bar graph representing the percentage of centralized nuclei in SVD + SBI PP7 and PP21 tissue samples. Preg pregnant, PP1 Postpartum day 1, PP3 Postpartum day 1, PP5 Postpartum day 5, PP7 Postpartum day 7, PP21 Postpartum day 21, IgGs Imunoglobulins G, DAPI 4′,6-diamidino-2-phenylindole, eMyHC embryonic myosin heavy chain, SVD spontaneous vaginal delivery, SBI simulated birth injury.

Previously, we had evaluated the impact of spontaneous vaginal delivery (SVD) and vaginal balloon distention, a well-established model of simulated birth injury (SBI), on the rat pelvic skeletal muscles. We had determined that SVD and vaginal distention with 3 mL balloon volume, which corresponds to the size of a term neonatal rat, do not induce sarcomere hyperelongation—the major cause of mechanical muscle injury in pubocaudalis (PCa; the most translationally relevant component of the rat PFM complex^23,26^) of late pregnant animals^26^. The above suggests that SVD does not induce this type of PFM injury in the rat model, as was also evident from the intrapartum sarcomere length measurements^26^. To confirm these findings using additional outcomes, we assessed PCa morphology after SVD on post-partum day 1 (PP1), PP3, PP5, PP7, and PP21. We deployed immunoglobulin G (IgG) and anti-embryonic myosin heavy chain (eMyHC) staining to identify the presence of damaged and regenerating myofibers, respectively (Fig. 1a). We also quantified the amount of central nucleation at the same time points as a second measure of muscle regeneration (Fig. 1a). After screening all tissue sections, we did not observe any IgG^+^ or eMyHC^+^ myofibers in any of the analyzed groups, supporting the notion that pregnancy and SVD do not induce injury of the rat PCa (Fig. 1b, c). Consistently, the amount of centrally nucleated myofibers remained constant throughout the post-partum period examined (Fig. 1d). The above provided us with an opportunity to evaluate the effects of lactation on skeletal muscle homeostasis after SVD without the confounding impact of birth injury.

To determine the effect of lactation on PCa regeneration in the setting of birth injury, we relied on the intrapartum SBI (SVD + SBI) model, using well-established methods (Fig. 1e)^27^. We assessed IgG^+^, eMyHC^+^ and centrally nucleated myofibers at 7 and 21 days after SVD + SBI. Both IgG^+^ and eMyHC^+^ myofibers were identified on PP7, highlighting the presence of both damaged and regenerating myofibers at early time points after birth injury (Fig. 1f, g). By day 21 after SVD + SBI, the IgG^+^ and eMyHC^+^ myofibers were no longer evident (Fig. 1f, g); however, the percentage of myofibers with centralized nuclei remained high (Fig. 1h), suggesting that the PCa muscle remodeling is ongoing. Employing both models (SVD and SVD + SBI), we were able to discriminate between the effect of lactation on muscle homeostasis vs muscle regeneration, as detailed below.

### Muscle stem cells are activated by spontaneous vaginal delivery (SVD)

**Fig. 2:**
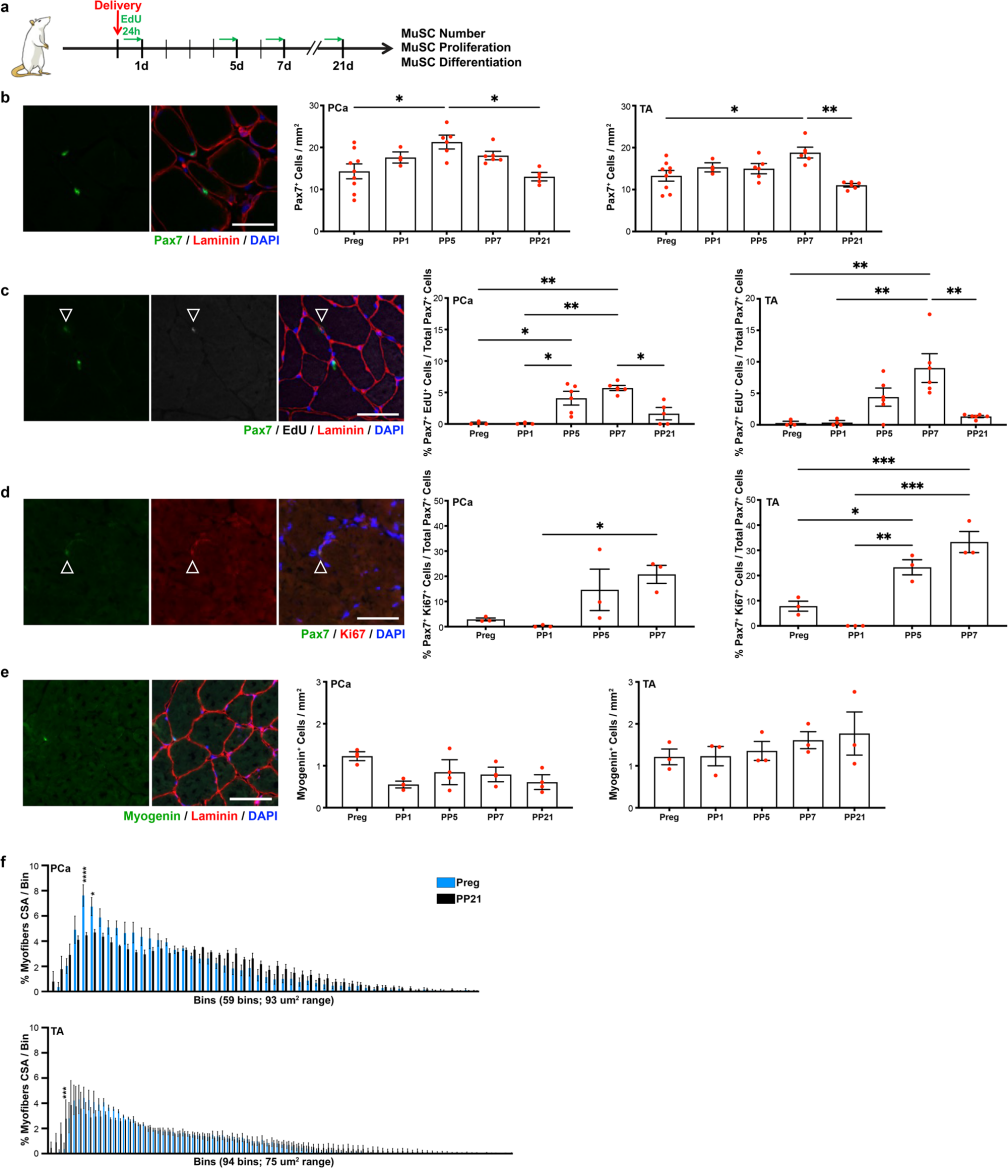
Muscle stem cell proliferation increases after spontaneous vaginal delivery. **a** Experimental summary for (**b**, **e**). **b** On the left, example of immunofluorescent staining against Pax7; on the right, bar graphs representing quantification of Pax7^+^ cells (MuSCs). Preg (*n* = 8, 9), PP1 (*n* = 4), PP5 (*n* = 6), PP7 (*n* = 6), and PP21 (*n* = 5-6). **c** On the left, example of immunofluorescent staining for against Pax7 and EdU; on the right, bar graphs representing quantification of Pax7^+^EdU^+^ cells (proliferating MuSCs). Preg (*n* = 4), PP1 (*n* = 4), PP5 (*n* = 6), PP7 (*n* = 5, 6), and PP21 (*n* = 5–6). **d** On the left, example of immunofluorescent staining for against Pax7 and Ki67; on the right, bar graphs representing quantification of Pax7^+^Ki67^+^ cells (proliferating MuSCs). Preg (*n* = 3), PP1 (*n* = 3), PP5 (*n* = 3), PP7 (*n* = 3). **e** On the left, example of immunofluorescent staining against myogenin; on the right bar graphs representing quantification of myogenin^+^ cells (differentiating cells). Preg (*n* = 3), PP1 (*n* = 3), PP5 (*n* = 3), PP7 (*n* = 3), PP21 (*n* = 3, 4). **f** Graphical representation of myofiber cross-sectional area distribution in pregnant and PP21 animals. Top graph refers to PCa and bottom graph - to TA. Preg (*n* = 3), PP21 (*n* = 3, 4). Full statistic info in Supplementary Table 1. For (**b**–**e**), PCa is on the left, TA is on the right. Scale Bar: 50 mm. Pax7 paired box 7, DAPI 4′,6-diamidino-2-phenylindole, PCa pubocaudalis, TA Tibialis Anterior, Preg pregnant, PP1 Postpartum day 1, PP5 Postpartum day 5, PP7 Postpartum day 7, PP21 Postpartum day 21, EdU 5-ethynyl 2´-deoxyuridine, eMyHC embryonic myosin heavy chain, CSA cross sectional area.

We first sought to determine MuSC behavior after SVD in PCa and non-pelvic hind limb muscle, tibialis anterior (TA) in non-lactating animals. Shortly after delivery, pups were removed. Rats received intraperitoneal 5-ethynyl 2´-deoxyuridine (EdU) injection 24 h before euthanasia, followed by immediate tissue harvest on day 20 of pregnancy, PP1, PP5, PP7 or PP21 (Fig. 2a). The early time points were selected to ascertain the acute muscle response, while the 21-day timepoint represents the end of the 3-week lactation period in rats. We observed a significant increase in MuSC reservoir in PCa on PP5 compared to pregnant animals at the end of gestation, despite SVD not causing PFM injury. In addition, we observed an expanded MuSC reservoir in TA on PP7 (Fig. 2b). The rise in cell number at these time points was driven by increased MuSC proliferation, evident from significantly higher proportion of Pax7^+^EdU^+^ cells in both muscles relative to the antepartum baseline (Fig. 2c). By the PP21, the MuSC number and proliferation returned to the level observed in late pregnancy (Fig. 2b, c). To further validate this finding, we also assessed MuSC expression of Ki67, another marker of cell proliferation. Consistent with the increase in EdU incorporation, we again observed a significant surge in cell proliferation in the acute PP period in PCa and TA (Fig. 2d). Given that MuSC proliferation was increased within the first 5 days after SVD, we went on to assess MuSC differentiation, using myogenin. Interestingly, the number of myogenin^+^ cells did not differ at any PP time point examined compared to that in pregnancy in either PCa or TA, maintaining baseline expression levels (Fig. 2e). To determine whether increased MuSC proliferation leads to muscle hypertrophy, we assessed fiber cross sectional area (CSA), comparing pregnant and PP21 tissues. We observed significant decrease in the number of small myofibers on PP21in PCa (Fig. 2f, top). A similar trend that did not reach statistical significance was observed in TA, where larger myofibers were present on PP21 (Fig. 2f, bottom).

Taken together, these results show that despite not causing an overt PFM injury, SVD induces a sharp increase in MuSC proliferation in the immediate PP period, resulting in larger myofibers. Importantly, MuSC proliferation was increased in both pelvic and non-pelvic muscles, suggesting that systemic changes govern the acute responses of skeletal muscles to fetal delivery.

### Lactation blocks muscle stem cell proliferation after spontaneous vaginal delivery

**Fig. 3:**
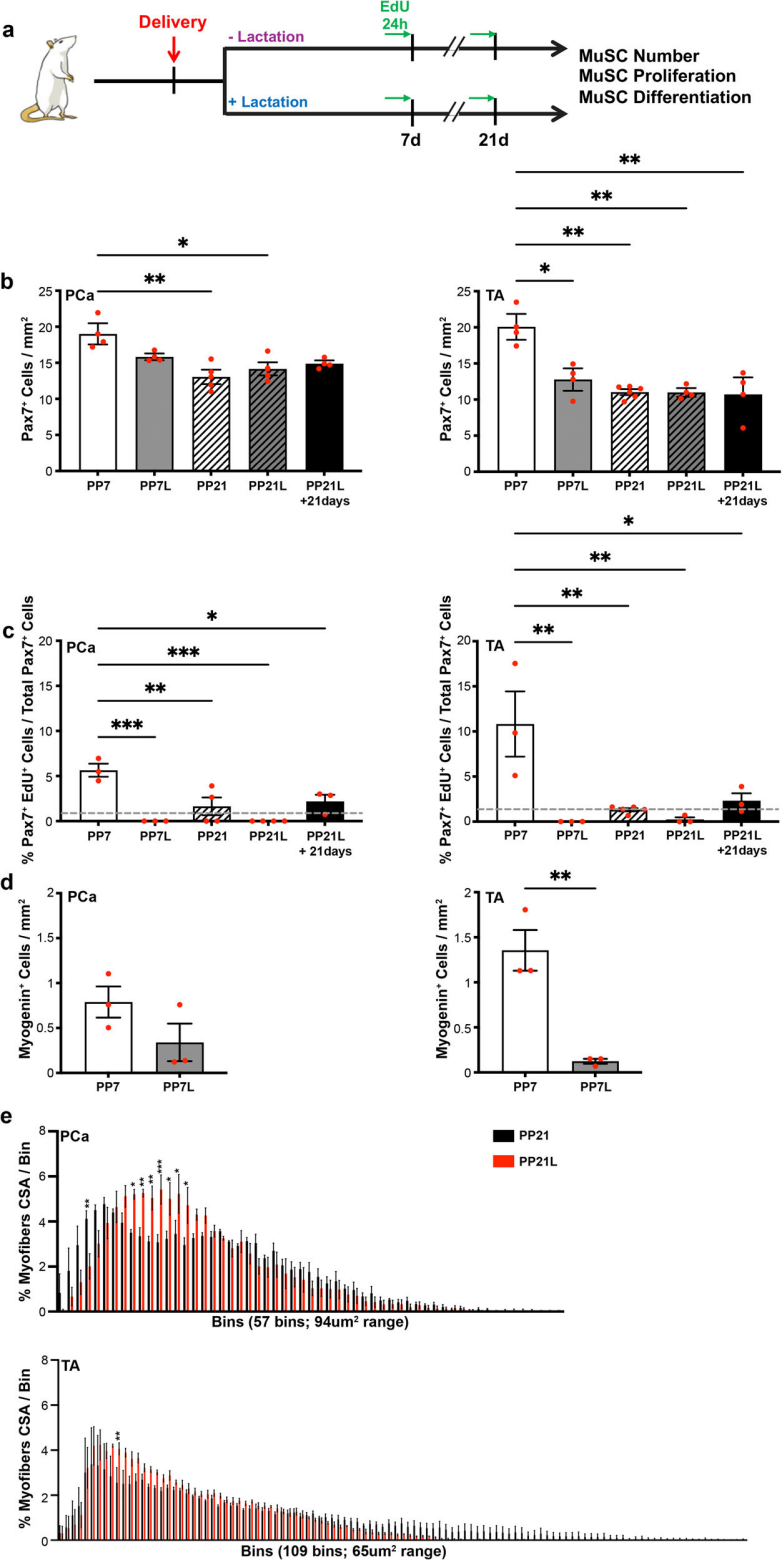
Lactation blocks muscle stem cell activation in the post-partum period following spontaneous vaginal delivery. **a** Experimental summary for (**b**–**e**). **b** Bar graph representing quantification of Pax7^+^ cells (MuSCs). PP7 (*n* = 4); PP7L (*n* = 4); PP21 (*n* = 5); PP21L (*n* = 4); PP21L+21days (*n* = 4). **c** Bar graph representing quantification of Pax7^+^EdU^+^ cells (proliferating MuSCs). Dotted line in gray represents the baseline level of proliferative MuSCs in non-pregnant unperturbed animals. PP7 (*n* = 3); PP7L (*n* = 3); PP21 (*n* = 4, 5); PP21L (*n* = 4); PP21L+21days (*n* = 3). **d** Bar graph representing quantification of myogenin^+^ cells (differentiating cells). PP7 (*n* = 3); PP7L (*n* = 3). **e** Graphical representation of the myofiber cross-sectional area distribution in pregnant, PP21, and PP21L groups. Top graph refers to PCa and bottom graph to TA. PP21 (*n* = 3); PP21L (*n* = 3). Full statistic info in Supplementary Table 2. For (**b**–**d**), PCa is on the left, TA is on the right. PCa pubocaudalis, TA Tibialis Anterior, PP7 Postpartum day 7, PP7L Postpartum day 7 lactating, PP21 Postpartum day 21, PP21L Postpartum day 21 lactating, CSA cross sectional area.

It is well known that during the lactation period major changes in the hormonal milieu^28^ and relocation of energy resources occur to support milk production^2,8^. Thus, we hypothesized that lactation would dampen MuSC activation and proliferation in response to SVD compared to non-lactating rats. To test this hypothesis, we randomly divided animals into non-lactating (pups removed) and lactating (rats housed with their pups) groups immediately after delivery. The MuSC number and proliferative ability were compared between the groups on PP7 and PP21 (Fig. 3a). The MuSC number did not differ between non-lactating vs lactating animals at either time point in PCa. However, the number of Pax7^+^ MuSCs in TA was significantly lower in lactating compared to non-lactating rats on PP7, indicating that lactation inhibited MuSC pool expansion observed in non-lactating animals (Figs. 2b and 3b). MuSC number did not differ at PP21 between lactating and non-lactating groups, and in both conditions, the reservoir was similar to the pregnancy levels, suggesting that the MuSC number returned to homeostatic levels (Figs. 2b and 3b). The robust EdU incorporation by proliferating MuSCs, observed in the non-lactating group, was notably absent in both PCa and TA in lactating animals on PP7 (Fig. 3c). On PP21, MuSCs failed to incorporate EdU in 100% of the PCa samples and in 75% of the TA samples in the lactating animals. MuSC proliferation in the non-lactating group returned to baseline (indicated by the gray dotted line) at this timepoint (Fig. 3c). To determine whether MuSC proliferation returns to baseline after a period of non-lactation, we housed the lactating animals for an additional 21 days after weaning the pups (PP42). MuSCs in both PCa and TA returned to basal proliferative levels at PP42, indicating that the inhibitory effect of lactation on MuSCs is reversable (Fig. 3c). Similarly, lactation significantly decreased the number of myogenin^+^ cells relative to the basal levels in TA, with a downward trend observed in PCa (Fig. 3d).

Given the observed effects of lactation on MuSC function, we next asked whether absence of MuSC proliferation throughout lactation negatively impacts muscle maintenance. To this effect, we compared myofiber size distribution at 21 days after vaginal delivery between animals who did vs did not lactate. PCa myofiber size distribution was enriched with myofibers ranging between 1082 and 1740 mm^2^ in animals that nursed their pups compared to non-lactating ones (Fig. 3e, top). Similar enrichment in myofibers ranging between 945 and 1010 mm^2^ was observed in TA muscle (Fig. 3e, bottom).

Taken together the above results show that in lactating animals, both PCa and TA MuSC numbers do not increase compared to the late pregnancy levels, and that proliferation and baseline differentiation of MuSCs are significantly reduced following SVD. Specifically, proliferative reduction is maintained throughout the duration of lactation. Consistently with the absence of MuSC activation in lactating conditions, we observed a reduction in the myofiber size at PP21 timepoint in lactating compared to non-lactating animals. Interestingly, lactation-induced changes affected both pelvic and limb muscles, suggesting that systemic cues are likely driving the observed phenotypes.

### Simulated maternal birth injury ablates the negative effect of lactation on muscle stem cell proliferation

**Fig. 4:**
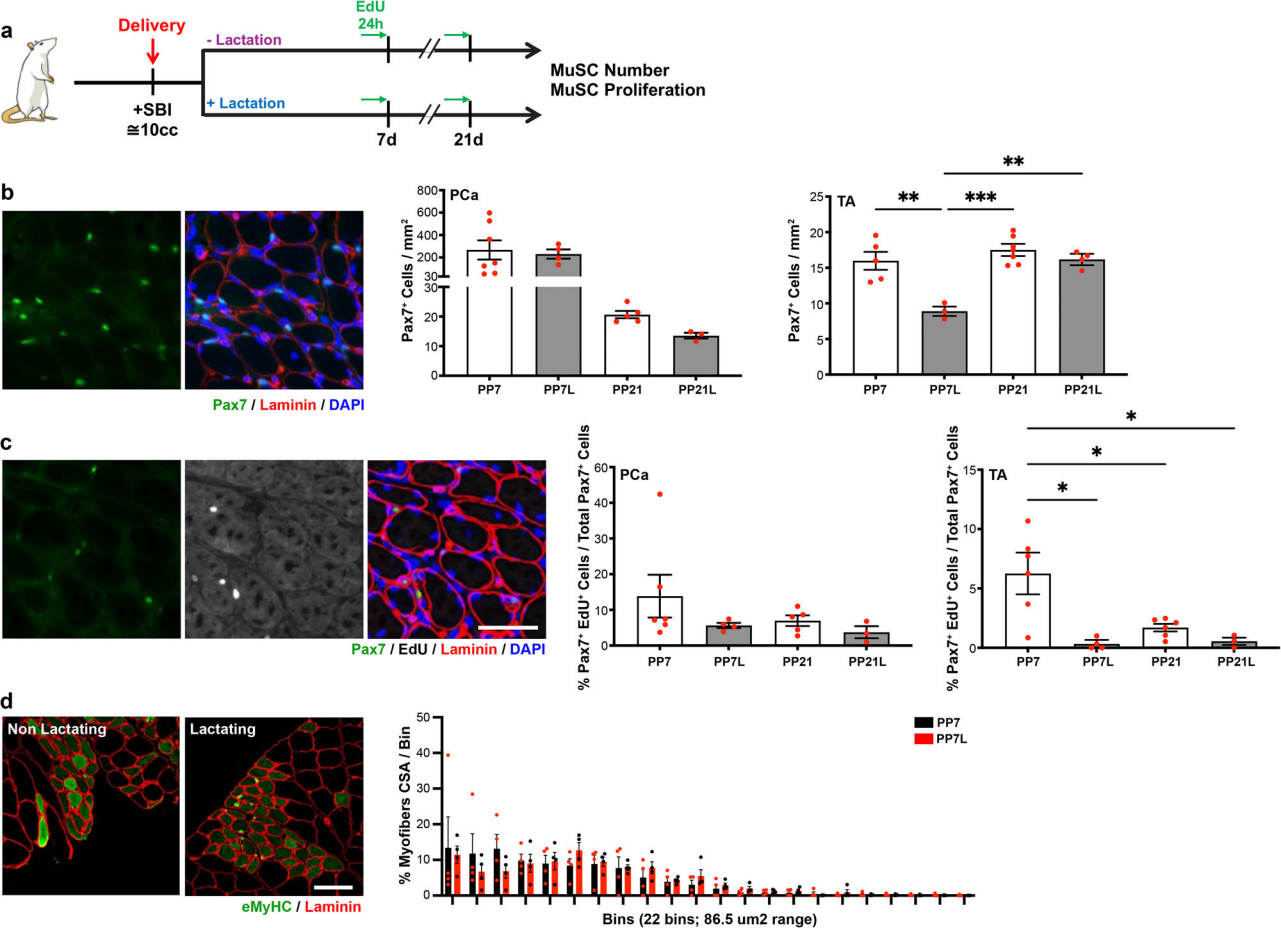
Simulated birth injury negates the inhibitory effect of lactation on pelvic muscle stem cells. **a** Experimental summary for (**b**–**d**). **b** On the left, example of immunofluorescent staining against Pax7; on the right, bar graphs representing quantification of Pax7^+^ cells (MuSCs). PP7 (*n* = 5,7); PP7L (*n* = 3,4); PP21 (*n* = 5,7); PP21L (*n* = 3,4). **c** On the left, example of immunofluorescent staining against Pax7 and EdU; on the right, bar graphs representing quantification of Pax7^+^EdU^+^ cells (proliferating MuSCs). PP7 (*n* = 6); PP7L (*n* = 4); PP21 (*n* = 5,6); PP21L (*n* = 3). **d** On the left, example of immunofluorescent staining against eMyHC; on the right, graphical representation of myofiber cross-sectional area distribution in PP7 and PP7L animals. PP7 (*n* = 3); PP7L (*n* = 3). For (**b**–**d**), PCa is on the left, TA is on the right. SBI simulated birth injury, DAPI 4′,6-diamidino-2-phenylindole, Pax7 paired box 7, PCa pubocaudalis, TA Tibialis Anterior, PP7 Postpartum day 7, PP7L Postpartum day 7 lactating, PP21 Postpartum day 21, PP21L Postpartum day 21 lactating, EdU 5-ethynyl 2´-deoxyuridine, eMyHC embryonic myosin heavy chain, CSA cross sectional area.

To determine whether the effect of lactation on MuSC function varies between animals who experienced vaginal delivery with and without birth injury, we utilized an established vaginal balloon distention SBI model^27^. SBI was performed intrapartum, after the delivery of 1–2 pups was observed. Following SVD + SBI, animals were randomly assigned to either lactating or non-lactating group (Fig. 4a). Rats were housed for either 7 or 21 days postpartum. As expected, 7 days following SVD + SBI, MuSC reservoir expanded dramatically in PCa, reaching Pax7^+^ MuSC density 10-fold higher than that observed on PP7 after vaginal delivery without birth injury (Figs. 2b, 3b, and 4b). The Pax7^+^ MuSC density in TA on PP7 was comparable between animals who did vs did not experience birth injury at the time of vaginal delivery (Figs. 2b, 3b, and 4b). The number of Pax7^+^ cells in the injured PCa did not differ in lactating compared to non-lactating animals exposed to SVD + SBI (Fig. 4b), whereas we observed a decrease in MuSC number in TA in lactating group, consistent with the results observed after SVD without injury (Figs. 3b and 4b). We then examined the proliferative response of MuSCs to SVD + SBI. In contrast to the lactation-induced suppression of PCa MuSC proliferation observed after SVD without injury (Fig. 3c), the proportion of Pax7^+^EdU^+^ cells in PCa did not differ between lactating and non-lactating groups 7 days following SVD + SBI (Fig. 4c). However, in the TA muscle that was not directly affected by birth injury, MuSC proliferation was decreased in lactating compared to non-lactating animals, similarly to what we observed in uninjured conditions. To further examine whether lactation affects PCa regenerative capacity in the presence of birth injury, we assessed the cross-sectional area of regenerating myofibers, identified by eMyHC. Consistently with the lack of difference in cell number and cell proliferation in response to SVD + SBI, eMyHC^+^ myofibers were no different in PCa of lactating relative to non-lactating animals at PP7 (Fig. 4d).

Taken together, these data suggest that in muscles subjected to direct injury, lactation-associated reduction in MuSC proliferation is overcome, and muscle regeneration proceeds efficiently. However, if the muscle is not directly injured, MuSC response to parturition is altered by the systemic milieu associated with lactation.

## Discussion

In this novel study, we assessed the impact of lactation on MuSC function in homeostatic and regenerative conditions. Furthermore, we compared the effects of lactation between muscles that were and were not directly impacted by vaginal delivery. With respect to the pelvic floor skeletal muscles, we examined the PCa part of the rat levator ani, homologous to the pubovisceralis portion of the human levator ani, both of which sustain the larger strains during parturition in the rat and the human, respectively^12^. For the non-pelvic control, we selected TA—a hind limb muscle commonly studied in rodents by researchers in the broader skeletal muscle field. The direct comparisons between PCa and TA allowed us to assess the effect of systemic factors associated with lactation uncoupled from direct muscle perturbation. We further capitalized on our findings that the rat PFMs do not experience detectable mechanical injury during SVD (Fig. 1a), but do so in a well-established rat SBI model (Fig. 1b) to compare the impact of lactation in these different scenarios.

We showed that the reservoir and the proliferative capacity of Pax7^+^ MuSCs increase in response to vaginal delivery (compared to late pregnancy), indicating that systemic changes in the early postpartum period affect MuSC behavior in both pelvic and non-pelvic muscles. Lactation impacts MuSCs by inhibiting cellular proliferation and reducing myogenic progression in both pelvic and non-pelvic appendicular muscles. These findings point towards the existence of systemic factors associated with lactation that regulate MuSC state. However, when significant direct injury, such as mechanical birth injury, is imposed on a muscle, MuSC behavior is indistinguishable between lactating and non-lactating animals. The above findings suggest that local cues released in response to birth injury overcome the inhibitory systemic effects of lactation on MuSCs of the PFM. However, MuSC reservoir and proliferative function of the non-pelvic TA muscle, were reduced in lactating compared to non-lactating animals following vaginal delivery with simulated birth injury counterparts, as was observed following vaginal delivery without birth injury. Notably, the current data indicate that the systemic factors associated with lactation override previously shown activating effect of factors released systemically by an injured muscle on MuSCs in skeletal muscles remote from the site of direct muscle injury^29^.

Lack of evident myofiber injury or regeneration after SVD, (Fig. 1a–d) coupled with increased EdU incorporation in MuSCs, and followed by the increase in the PCa and TA myofiber size (Fig. 2c, f), raises an important question: how do MuSCs become activated? Activation of MuSCs in the absence of myofiber injury and in non-pathological conditions has been previously reported in studies evaluating muscle response to eccentric and concentric exercise^30,31^. For instance, treadmill downhill running causes activation of MuSCs in the rat extensor digitorum longus muscle that histologically did not demonstrate myofiber injury^30^. The amount of activated cells, however, was reported to be smaller compared to soleus muscle that had myofiber degeneration after the same exercise^30^. Muscle biopsies isolated from men undergoing 14 days of endurance training, showed increased number of MuSCs and increased CSA of Type IIA myofibers in the absence of any notable injury^31^. These studies demonstrate that activation of MuSCs in the absence of clear histological markers of injury is possible. This phenomenon could be driven by mechanical cues such as mitogenic factors released by myofibers or extracellular matrix (ECM)^30,32^. Another study proposed that hepatocyte growth factor (HGF), which increases in serum following exercise, could be driving MuSC activation in response to exercise^33^. Indeed, it has been extensively demonstrated that muscle stretch induces release of HGF from the skeletal muscle ECM driving myoblast and MuSC activation^34–36^. Given that mechanical cues in the absence of muscle injury are involved in MuSC activation in the context of exercise, similar pathways could potentially underlie the activated MuSC phenotype observed in the rat skeletal muscles in response to parturition. This possibility is especially intriguing as HGF has been shown to increase in women during pregnancy^37,38^. Deciphering whether HGF or similar cues are released in response to stretch caused by SVD, leading to activation of MuSCs in PCa and TA in the absence of muscle injury, is an important direction for future studies and development of novel therapies for muscle atrophy.

While further investigation is needed to understand what stimuli drive MuSC activation in the early post-partum period in both PCa and TA, we demonstrate that this activation leads to increased myofibers size 21 days after vaginal delivery in non-lactating animals (Fig. 3c). Furthermore, we discovered that MuSC proliferative capacity is drastically reduced in pelvic and limb muscles in response to lactation, with EdU incorporation below the basal level observed in unperturbed non-pregnant animals. Strikingly, when pups are weaned from their mother, MuSC proliferation returns to the homeostatic level within 21 days. Drastic hormonal changes and relocation of energy resources towards milk production in lactating animals likely account for this systemic impact of lactation.

Multiple studies looked at the hormonal milieu of lactating rats. Progesterone has been shown to increase immediately after delivery, peaking at around 10 days of lactation and then decreasing back to basal levels by day 21, with the drop in progesterone observed as soon as the pups are separated from the mother^39–41^. Similarly, prolactin levels start increasing around PP2, peaking on PP7, and then decreasing until weaning^41^. Oxytocin increases during parturition and remains elevated (compared to pregnancy levels) immediately after delivery^42^. During lactation, both prolactin and oxytocin levels are cyclical and dependent on the pups’ suckling^43,44^. Estradiol is low for the first 10 days of lactation and then slowly goes back to pre-pregnancy baseline levels by day 21^39^. No differences in testosterone levels have been detected throughout lactation^39^. To the best of our knowledge, no information is available on hormonal levels in non-lactating rats after delivery, thus a direct comparison is not currently possible.

Energy relocation during lactation has been studied in multiple farm animals, demonstrating increased skeletal muscle catabolism, detected by the systemic and tissue-level rise in free amino acids or by quantification of skeletal muscle total proteins^8,45,46^. Consistently, transcriptomic studies in the lactating rat model show decrease in the expression of genes associated with protein transcription and translation, suggesting reduction in protein synthesis in lactating vs cycling animals^47^. The increased catabolism and decreased protein synthesis lead to skeletal muscle atrophy, affecting different muscle groups with variable severity^6,7,45,48^. In addition, to facilitate milk production, metabolic changes occur in maternal skeletal muscles, specifically in muscles enriched for glycolytic myofibers, through reduction of basal levels of glucose uptake and increase in insulin resistance^49^. With these findings in mind, it is possible that the reduction in the myofiber size observed in lactating relative to non-lactating animals in the current study (Fig. 3e) is driven by a combinatorial effect of protein catabolism and reduction in MuSCs proliferation. Further studies are needed to delve into the mechanisms that underly this fascinating physiology.

When maternal birth injury is sustained at the time of delivery, the negative effect of lactation on MuSC proliferation is completely abrogated in the directly injured PCa muscle but not in the appendicular TA muscle that is not directly affected by the birth injury (Fig. 4b, c). These novel results indicate that muscle response to direct mechanical injury negates the systemic effects of lactation on MuSCs, but that the systemic effect of muscle injury cannot overcome inhibitory lactation-associated signals in muscles remote from the site of injury.

Previous studies by Rodgers et al. had demonstrated that injury to a hind limb muscle induces MuSCs in the contralateral leg to assume a G_alert_ phenotype, which is characterized by the intermediate EdU incorporation and time to first division compared to quiescent and activated cells^29^. The G_alert_ MuSC phenotype has been shown to be promoted by HGF released from the muscle ECM in response to injury^29^. In the current study, in the absence of lactation, we identified increase in MuSC EdU incorporation in TA 7 days after SVD + SBI (7% Pax7^+^EdU^+^ out of all Pax7^+^cells) compared to that observed before delivery in pregnant animals (1% Pax7^+^EdU^+^ out of all Pax7^+^cells), Fig. 4c. This finding suggests potential for shared mechanisms that govern the current and previously identified G_alert_ MuSC phenotype. In the absence of lactation, surprisingly, we observed a similar increase in the MuSC EdU incorporation following vaginal delivery without overt muscle injury (Fig. 2d). In contrast, lactation inhibits MuSC proliferation in TA following SVD with or without birth injury, suggesting that the effect of lactation is stronger than the systemic effect mediated by remote muscle injury. Further studies are needed to determine specific mechanisms that govern the impact of lactation on MuSCs.

In the current study, we demonstrate that the rat PFMs do not exhibit markers of myofiber injury after SVD allowing us to uncouple the effects of lactation from the impact of birth injury. Using the rat models of vaginal delivery with and without birth injury, we show that MuSCs behave differently in non-lactating compared to lactating animals following vaginal delivery without injury. In the absence of lactation, MuSCs become activated as early as 5 days after delivery; in contrast, postpartum MuSC proliferation is inhibited in lactating rats. This inhibitory effect of lactation on MuSC proliferation was observed in both pelvic and limb muscles, implicating the role of systemic factor(s). In addition, we show that the inhibitory effect of lactation on MuSCs is negated by the cues released from the PFMs subjected to birth injury. However, the systemic effect of muscle injury on MuSCs in the muscles not directly impacted by injury is modulated by the lactation status. These novel findings open fruitful avenues for the future studies focused on the identification of systemic factor(s) capable to modulating MuSC proliferation, in turn, applying this knowledge to enhance PFM regeneration after birth injury in women, and, more broadly, to all skeletal muscles.

## Methods

### Animals

All procedures were approved by the University of California, San Diego Institutional Animal Care and Use Committee and performed at the designated animal facilities. Animals were housed in humidity and temperature-controlled environment with access to food and water *ad libitum*. A total of 61 pregnant (Day 20/23) 3-month-old female Sprague-Dawley rats (Envigo, Indianapolis, IN) were employed for all experiments. Animals, randomly assigned (random number generator) to non-lactating (pups removed) or lactating groups following SVD, were euthanized by CO_2_ inhalation followed by thoracotomy on PP1, PP5, PP7, or PP21. Similarly, animals that underwent SBI at the time of parturition were randomly divided into non-lactating and lactating groups and euthanized by the same method on PP7 or PP21. All animals received an intraperitoneal injection of EdU 24 h before tissue harvest (5 mg/g of body weight). Age-matched pregnant animals (day 20) were used as controls. Bilateral PCa, and non-pelvic control muscle, TA, were harvested immediately following euthanasia. None of the animals were excluded from this study. Number of pups per animal was not accounted for in the analysis.

### Simulated birth injury (SBI)

Simulated birth injury was performed using a well-established vaginal balloon distention protocol^27^. Briefly, after delivery of 1–2 pups, anesthesia was induced with 4% isoflurane with oxygen and maintained using 2% isoflurane for the rest of the procedure. A 12-French transurethral balloon catheter (Bard Medical, Covington, GA) with the tip cut off was inserted into the vagina and a 130 grams weight was attached to the end of the catheter. The balloon was inflated to 10 ml and left in place for 2 h, after which, the balloon was deflated to 5 ml and pulled through the introitus to simulate the circumferential and downward distention associated with fetal crowning and parturition.

### Immunofluorescent staining

PCa and TA were cryosectioned into 10 µm cross-sections.

For IgG staining tissues were fixed using 2% paraformaldehyde (PFA), washed in PBS, and incubated in blocking buffer (10% goat serum in PBS) for 1 h. Slides were then incubated with primary anti-laminin antibody (Millipore Sigma; L9393; 1:200) for 2 h at room temperature, washed and incubated with secondary antibodies (Alexa Fluor 546 goat anti-rabbit IgG (Invitrogen; cat#: A11035) and Alexa Fluor plus 488 goat anti-rat IgG; Invitrogen; A48262) for laminin and IgGs respectively.

For Embryonic myosin heavy chain (eMyHC)staining, slides were fixed with 100% cold acetone, washed in PBS and incubated with blocking buffer (20% goat serum + 0.3% Triton X-100 in PBS) for 1 h before incubation with primary anti-laminin antibody and eMyHC (DSHB; cat#: F1.652; 1:100) in blocking buffer, overnight. After washing in PBS, slides were then incubated with secondary antibodies (Alexa Fluor 546 goat anti-rabbit IgG and Alexa Fluor 488 goat anti-mouse IgG (Invitrogen; cat#: A11029)) for 2 h.

For Pax7, Ki67, and myogenin staining, slides were fixed with 4% PFA, washed in PBS and incubated with blocking buffer (20% goat serum + 0.3% Triton X-100 in PBS) for 1 h before incubation with primary anti-laminin antibody in blocking buffer, overnight. Slides were then washed and incubated with secondary antibody (Alexa Fluor 546 goat anti-rabbit IgG) for 1 h, washed with PBS, and treated with EdU reaction (prepared using the manufacturer protocol) for 30 min. Slides were then post-fixed with 4% PFA solution before treatment with Antigen Retrieval Solution in a boiling water bath for 15 min. Slides were then washed in PBS, incubated in blocking buffer before overnight incubation with either Pax7 (DSHB; cat#: Pax7-c; 1:100), ki67 (abcam; ab15580; 1:100), or myogenin (BD Pharmingen; cat#: 556358; 1:100) antibodies. After washing, slides were then incubated with secondary antibody (Alexa Fluor 488 goat anti-mouse IgG) for 1 h.

All slides were then incubated with DAPI (Thermo Scientific; cat#: 62248) at a 1–1000 dilution to identify nuclei and mounted with Fluoromount and a coverslip.

### Quantification

For IgG slides, one slide per animal (containing sections collected throughout the whole muscle length) was scanned and all sections were analyzed for the presence/absence of IgG^+^ myofibers. One section (the largest one) was selected for quantification of centralized nuclei. Images were not modified before quantification.

For Pax7, EdU, Ki67, and Myogenin staining, three random images per each PFM section from both right and left sides, and four random images per each section of TA were taken at 20X magnification by an investigator blinded to the group assignment. Images were overlayed and analyzed using GIMP or Photoshop 2022. Cells positive for each marker of interest were manually identified and counted. Co-staining was performed for Pax7 and EdU and for Pax7 and Ki67. Pax7^+^ and myogenin^+^ cells were quantified per unit area. Double positive Pax7^+^EdU^+^ or Pax7^+^Ki67^+^ cells were quantified as a percentage of all Pax7^+^ cells. Images were not modified before quantification.

To measure myofibers CSA, for each biological replicate, a stitched image of the full muscle tissue section was taken for PCa (bilateral muscles were harvested and analyzed) and TA (either right or left muscle per animal was harvested and analyzed). ImageJ custom macro was used for myofiber identification. Cross-sectional area was obtained using ImageJ. Images were not modified before quantification.

To quantify Embryonic Myosin Heavy Chain (eMyHC) slides, for each PFM a full image of the tissue section from each side was taken at 10x magnification. ImageJ was used to assess eMyHC^+^ myofiber CSA. Images were not modified before quantification.

All slides were imaged using either Keyence BZ-X810 (Objective used: BZ Objective Lens (20x Phase) Plan Fluorite 20x LD PH) or Nikon Eclipse Ti2 microscopes (Objective used: CFI60 Plan Apochromat Lambda D 10x Objective Lens, N.A. 0.45, W.D. 4.0 mm, F.O.V. 25 mm, DIC).

### Statistical analysis

Data were analyzed using GraphPad Prism v10, San Diego, CA. *P* < 0.05 were considered significant. For analysis of MuSC number, percentage of proliferating MuSCs, and myogenin^+^ cell number, one-way analysis of variance (ANOVA) followed by post hoc pairwise comparisons using Tukey’s multiple comparison test were used. Mean myofiber CSA was compared between groups using Student *t* test. Myofiber size distribution was obtained by first eliminating the top and the bottom 1% and subsequently determining the number of bins necessary to describe the distribution (square root of the number of myofibers quantified per biological replicate). To obtain the range for each bin, the bin number was divided by the difference between the maximum and the minimum values. The two conditions were then compared per each bin using two-way ANOVA followed by post hoc pairwise comparisons using Sidak multiple comparison test.

## Supplementary information

Supplementary Data 1

Supplementary Table 1

Supplementary Table 2

## Data Availability

Data are provided within the manuscript. Raw data are available upon request.
